# The Sufferings of Christ

**Published:** 1887-10-01

**Authors:** 


### WORDS OF CONSOLATION.

[Communications for this column should be addressed, "Chaplain," care of the Editor of THE HOSPITAL, who will gratefully welcome any suggestions or inquiries from matrons, nurses, and the staff generally.]

### LII.—THE SUFFERINGS OF CHRIST.

(*For Reading to the Sick.*)

ONE of the saddest tokens of our fallen state is the length of time it takes before we recognise in our personal gifts or station, in the circumstances and complications of our life, in our difficulties and trials, in our relations to others or to the world,—the will of God, as revealed to us, teaching us what we are, and for what we ought to live. "Why have I this to struggle with or to endure? Why am I singled out for a trial, while others are spared? How came I to be placed in such circumstances? Why do I suffer more than most people? I should be so different if God had been pleased to order a happier lot for me." Whenever such thoughts are given way to they show a will not yet bent down to God's purpose. The Agony for us will sometimes consist in the bending. "Years often pass away before our early dreams, like mists stealing up the mountain-side, clear away; before the end of our life becomes visible, and the several objects around us fall into their true relative position; before we set ourselves resignedly to fulfil the real objects for which we were sent into this world. What long periods of waste and rebellion often intervene before the very first and simplest truths regarding the end of our being come into view! How much of life is simply an aspiring after unrealities, or else a fretting and chafing against an irreversible will, which, if lovingly accepted, would be the true bond of union with Our Lord, the starting point of an unknown and boundless beatitude." (Canon Carter).

Again, as we are constrained to look suffering in the face, or it may be death, and we try to be resigned even as we pray "Thy will be done," we must not be surprised if our silent agony is but little perceived by some nearest to us. What if Peter, James, and John are sleeping! There may be intimate friends who are all that we could desire under ordinary circumstances; there may be some who have followed us into the very scene of our latest tribulation, who remain by our side and minister unto us without ever failing in their watchfulness as Our Lord's disciples did: and yet even these may not know nor understand at all the cost at which our resignation is accomplished. The Agony of Our Lord is a great mystery. He was in the shade; He was accompanied; He was ministered unto by an angel; and yet in one sense He suffered alone. His resignation was completed without any creature perceiving the depths He traversed. There is mystery, too, in each of our individual dispensations. The heart alone with God knoweth its own bitterness. When we are called upon to resign what most we prize, whether it be health, or life, or a long-cherished idea, or one who is more than life to us, those nearest may understand little or nothing of the sacrifice, as with strong crying and tears we pass through the waves, and out at last to the blessed calm remaining for those whose minds are stayed on God. Meanwhile, whatever the discipline ordained for the sacrifice of the will, our only safety must be found in sympathy with Jesus in the Garden. "By Thine Agony Good Lord deliver us"—from what? Surely from the confusion, the remorse, the bitter agony, from the hell which must otherwise be the portion of self-will following its own devices until it reach the terrible climax of permanent antipathy to the Will of God.

(*To be continued.*)

